# Sociodemographic patterning of dietary profiles among Inuit youth and adults in Nunavik, Canada: a cross-sectional study

**DOI:** 10.17269/s41997-022-00724-7

**Published:** 2022-12-08

**Authors:** Amira Aker, Pierre Ayotte, Chris Furgal, Tiff-Annie Kenny, Matthew Little, Marie-Josée Gauthier, Amélie Bouchard, Mélanie Lemire

**Affiliations:** 1 Axe santé des populations et pratiques optimales en santé, Centre de recherche du CHU de Québec-Université Laval, Québec, Québec Canada; 2 Département de médecine sociale et préventive, Université Laval, Québec, Québec Canada; 3 Centre de toxicologie du Québec, Institut national de santé publique du Québec, Québec, Québec Canada; 4 Department of Indigenous Studies, Trent University, Peterborough, Ontario Canada; 5 School of Public Health and Social Policy, University of Victoria, Victoria, British Columbia Canada; 6 Nunavik Regional Board of Health and Social Services, Kuujjuaq, Québec, Canada; 7 Institut de biologie intégrative et des systèmes (IBIS), Université Laval, Québec, Québec Canada

**Keywords:** Indigenous populations, Dietary patterns, Inuit, Traditional foods, Populations indigènes, habitudes alimentaires, Inuit, aliments traditionnels

## Abstract

**Objectives:**

Country (traditional) foods are integral to Inuit culture, but market food consumption is increasing. The *Qanuilirpitaa?* 2017 Nunavik Health Survey (Q2017) reported similar country food consumption frequency compared to that in 2004; however, examining food items individually does not account for diet patterns, food accessibility, and correlations between food items. Our objective was to identify underlying dietary profiles and compare them across sex, age, ecological region, and food insecurity markers, given the links among diet, health, and sociocultural determinants.

**Methods:**

Food frequency and sociodemographic data were derived from the Q2017 survey (*N* = 1176). Latent profile analysis identified dietary profiles using variables for the relative frequencies of country and market food consumption first, followed by an analysis with those for country food variables only. Multinomial logistic regression examined the associations among dietary profiles, sociodemographic factors, and food insecurity markers (to disassociate between food preferences and food access).

**Results:**

Four overall dietary profiles and four country food dietary profiles were identified characterized by the relative frequency of country and market food in the diet. The patterns were stable across several sensitivity analyses and in line with our Inuit partners’ local knowledge. For the overall profiles, women and adults aged 30–49 years were more likely to have a market food–dominant profile, whereas men and individuals aged 16–29 and 50+ years more often consumed a country food–dominant profile. In the country food profiles, Inuit aged 16–29 years were more likely to have a moderate country food profile whereas Inuit aged 50+ were more likely to have a high country food–consumption profile. A low country and market food–consumption profile was linked to higher prevalence of food insecurity markers.

**Conclusion:**

We were able to identify distinct dietary profiles with strong social patterning. The profiles elucidated in this study are aligned with the impact of colonial influence on diet and subsequent country food promotion programs for Inuit youth. These profiles will be used for further study of nutritional status, contaminant exposure, and health to provide context for future public health programs.

**Supplementary Information:**

The online version contains supplementary material available at 10.17269/s41997-022-00724-7.

## Résumé

**Objectifs:**

Les aliments traditionnels font partie intégrante de la culture inuite, mais la consommation d’aliments du marché est en augmentation. L’enquête de santé des Inuit *Qanuilirpitaa?* réalisée en 2017 (Q2017) a mis en évidence que la fréquence de consommation d’aliments traditionnels était similaire à celle rapportée en 2004. Or, les fréquences de consommation des aliments pris individuellement ne tiennent pas compte des habitudes alimentaires, de l’accessibilité des aliments et des corrélations entre les aliments consommés. Notre objectif était d’identifier les profils alimentaires sous-jacents et de les comparer selon le sexe, l’âge, la région écologique et les marqueurs d’insécurité alimentaire, étant donné le lien entre l’alimentation, la santé et les déterminants socioculturels.

**Méthodes:**

Les données sur les fréquences alimentaires et sociodémographiques sont issues de l’enquête Q2017 (N=1176). L’analyse des profils latents a permis d’identifier des profils alimentaires en utilisant les variables pour les fréquences relatives de la consommation d’aliments traditionnels et du marché et uniquement celles pour les aliments traditionnels. Des régressions logistiques multinomiales ont été utilisées pour examiner les associations entre les profils alimentaires, les facteurs sociodémographiques et les marqueurs d’insécurité alimentaire (pour dissocier les préférences alimentaires de l’accès aux aliments).

**Résultats:**

Quatre profils alimentaires globaux et quatre profils alimentaires spécifiques à la consommation d’aliments traditionnels ont été identifiés en fonction de la fréquence relative des aliments traditionnels et des aliments du marché dans l’alimentation. Les profils étaient en accord avec les connaissances locales de nos partenaires Inuit. Pour les profils alimentaires globaux, les femmes et les adultes âgés de 30 à 49 ans étaient plus susceptibles d’avoir un profil dominé par les aliments du marché, tandis que les hommes et les personnes âgées de 16 à 29 ans et celles de 50 ans et plus avaient plus fréquemment un profil dominé par les aliments traditionnels. En ce qui concerne les profils de consommation d’aliments traditionnels, les Inuit âgés de 16 à 29 ans étaient plus susceptibles d’avoir un profil modéré de consommation d’aliments traditionnels, tandis que les Inuit âgés de 50 ans et plus étaient plus susceptibles d’avoir un profil élevé de consommation d’aliments traditionnels. Un profil bas de consommation d’aliments traditionnels et de marché était associé à une prévalence plus élevée de marqueurs d’insécurité alimentaire.

**Conclusion:**

Nous avons identifié différents profils alimentaires et ces derniers étaient associés à des caractéristiques socio-démographiques distinctes. Les profils alimentaires mis en lumière dans cette étude concordent avec l’impact du colonialisme sur l’alimentation au Nunavik et aux programmes subséquents de promotion des aliments traditionnels auprès des jeunes Inuit. Ces profils seront utilisés pour une étude plus approfondie du statut nutritionnel, de l’exposition aux contaminants et des issues de santé afin d’identifier des pistes de solutions pour les futurs programmes de santé publique.

## Introduction

Foods harvested from the land, rivers, and sea, also known as country foods, are an integral part of Inuit culture, providing exceptional nutrition and contributing to overall health and well-being. Country food meats such as marine mammals, caribou, and seafood provide protein, essential minerals (iron and zinc), and vitamins (Blanchet et al., 2000; Fediuk et al., 2002; Gagné et al., 2012; Kuhnlein et al., 1991; Kuhnlein & Receveur, 2007; Kuhnlein & Soueida, 1992; Lawn & Harvey, 2003; Lemire et al., 2015). Meanwhile, fish and marine mammals are excellent sources of omega-3 fatty acids, selenium, and vitamins A and D (Andersen et al., 2013; Bang et al., 1980; Courraud et al., 2020; Kuhnlein & Soueida, 1992; Lemire et al., 2015).

However, various factors have led to changing dietary patterns in Inuit populations (Little et al., 2021). Colonization of the Arctic and the forced settlement of many of its Indigenous peoples have contributed to a decline in traditional country food consumption, and the effects of this colonization continue to impact food security and determinants of health among Inuit (Caughey et al., 2021; Egeland et al., 2011a; Lougheed, 2010; McCartan et al., 2020). Transportation and communication with southern regions have increased in the last several decades, leading to greater availability and consumption of market foods (Blanchet et al., 2007). Salaried employment reduced participation in traditional activities, as well as time available for hunting and fishing. The combined effects of climate change, population demographic changes, high cost of harvesting activities (equipment, transportation, etc.), and reduced food species density also made it more difficult to sustain a traditional lifestyle (Kuhnlein et al., 2004; Little et al., 2021; Nancarrow & Chan, 2010). Despite public health advisories stressing the importance of balancing the nutritional benefits with contamination risks, concerns regarding environmental contaminants may have also contributed to a decline in country food consumption (Calder et al., 2019; Krummel & Gilman, 2016; Laird et al., 2013; Lemire et al., 2015; Little et al., 2021). Further, market foods are more expensive in northern communities than in the south due, in part, to the elevated transportation costs of delivering goods to remote northern locations (Duhaime & Andrée, 2012), despite food subsidy programs that fail to account for retailers’ pricing practices (Galloway, 2017). All these compounding factors have been associated with a high prevalence of food insecurity in Arctic communities (Chan et al., 2006; Collings et al., 2016; Egelandet al., 2011b; Huet et al., 2012, 2017; Skinner et al., 2014) with more families relying on non-nutrient-dense market foods, particularly among children and young Inuit (Huet et al., 2017), leading to poor nutritional status and lower nutrient intake (Egeland et al., 2011b; Huet et al., 2012; Little et al., 2021).

Nunavik in northern Quebec, Canada, is home to approximately 14,000 Inuit (known as Nunavimmiut). As in other Arctic regions, shifts in dietary and lifestyle patterns alongside environmental changes and the inaccessibility of healthy market foods were observed between the 1992 Santé Québec Survey in Nunavik and the *Qanuippitaa?* Nunavik Inuit Health Survey in 2004. Consumption of country foods declined over that decade (1992–2004), but carbohydrate and sodium levels increased substantially, particularly among young Inuit aged 18–29 years who reported that sugary foods comprised the largest proportion of their total caloric intake in 2004 (Blanchet et al., 2007). Changes from traditional dietary consumption profiles in Indigenous communities to more “western”- or “southern”-based profiles including excessive non-nutrient-dense foods have been linked to increases in obesity, type-2 diabetes, cardiovascular disease, and unhealthy blood lipid profiles (Gracey & King, 2009; Kellett et al., 2012; Kuhnlein et al., 2004; Little et al., 2021; Sheikh et al., 2011). Several programs have been implemented at local and regional scales to promote Inuit culture and lifestyle, country foods, and a healthier market food diet, particularly among younger Inuit.

It is important to understand changing dietary patterns in the Inuit population given the link between dietary patterns, socio-environmental issues, and chronic disease (He et al., 2021; Marx et al., 2021; Pestoni et al., 2021) and the complex dynamic interplay between nutrients and contaminant exposure (Domingo, 2017; Landrigan et al., 2020; Sabaté et al., 2016). An update to the Nunavik Inuit Health Surveys (from 1992 and 2004) was conducted in 2017, and results of this latest survey suggest a modest increase in country food consumption since 2004 (Allaire et al., 2021). However, the types of country foods more frequently consumed seem to differ from 2004, and differences in consumption appeared across various subpopulations by sex, age, and ecological region. A limitation of these findings was the focus on *individual* food items and groups because isolating specific foods may not provide a wholistic picture of overall nutrition or dietary preferences and habits (Cespedes & Hu, 2015). A complementary approach to food-based analyses and recommendations may be to identify underlying dietary profiles that exist in the population, thereby accounting for correlations between different food items and the complexities of food accessibility and preferences. Our objectives for this study were to (1) identify overall dietary patterns including country and market food consumption among Nunavimmiut aged 16 years and above; (2) identify country food dietary patterns more specifically; and (3) compare the dietary profiles by sex, age, ecological region, and food insecurity markers in Nunavik.

## Methods

### Study population

**Fig. 1:**
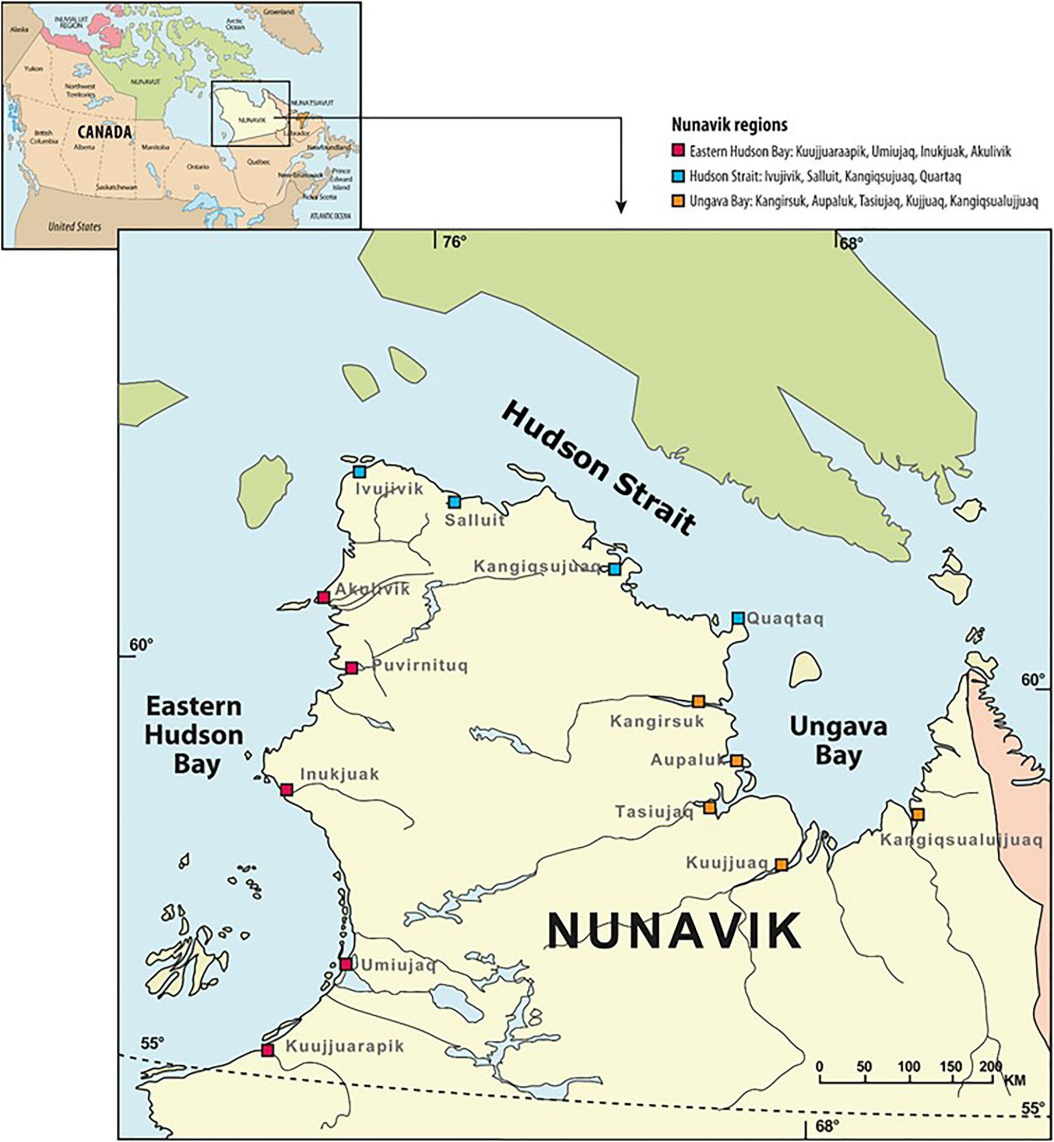
Map of Nunavik, northern Quebec, Canada

This study used data from the *Qanuilirpitaa?* 2017 Nunavik Inuit Health Survey (Q2017), a population health survey that assessed the health status of Nunavimmiut. Details on the Q2017 survey have been described elsewhere (Hamel et al., 2020). Briefly, the survey targeted the permanent residents of Nunavik aged 16 years and older and implemented a stratified proportional model for respondent selection. Stratification was based on region of residence and age category. A total of 1326 individuals participated in the survey from August 19, 2017 to October 5, 2017, from 14 villages settled along the ecological regions Ungava Bay, Hudson Strait, and Hudson Bay (Fig. 1). Data collection included questionnaires, clinical measurements, and human biological sampling of urine and blood.

Ethical approval was received from the Comité d’éthique de la recherche du Centre Hospitalier Universitaire de Québec - Université Laval (no. 2016-2499). The survey was conducted in close collaboration with several organizations from Nunavik (including but not limited to the Kativik Regional Government, Makivik Corporation, and the Nunavik Regional Board of Health and Social Services (NRBHSS)) and governed by the OCAP® principles (Ownership, Control, Access, and Possession). Furthermore, several meetings were held with the Data Management Committee (DMC), in which there was heavy representation of Inuit colleagues and partners, to discuss and interpret survey results, including the present study. The DMC and the Nunavik Nutrition and Health Committee (NNHC) provided an essential role in interpreting and providing context around the results for a thorough understanding of the complexities surrounding dietary patterns.

### Dietary assessment

Dietary patterns were assessed using a food frequency questionnaire (FFQ) that measured the consumption frequency of each item in the last 3 months but did not take serving sizes into consideration. The FFQ included traditional country foods and market foods (Online resource 1). Food items were rated on a scale of 1 (item consumed never or less than once a month) to 7 (item consumed four times or more a day). For the purposes of our analysis, five frequency categories were used: (1) never or less than once a month; (2) one to three times a month; (3) once a week; (4) two to six times a week; and (5) daily. The last two categories were dropped because several food items were not reportedly consumed more than once per day, and this was leading to problems with model convergence. Sensitivity analyses were performed using dichotomous dietary variables (below and above the median) to ensure model stability (data not shown).

### Food insecurity markers

The Q2017 survey included an adapted version of the USDA Food Security Survey Module (United States Department of Agriculture (USDA), 2012) questions on food security. Questions were asked at the individual scale and focused on food access during the 12 months prior to the survey. We opted to use specific food security constructs rather than the composite food security classification for our analysis because the food security classification scheme incorporates questions on food preferences and food access not directly relevant to our study aims (Furgal et al., 2022; Teh et al., 2017). To disassociate food preferences from food access, the following food insecurity questions were assessed separately, all of which were answered by “Yes,” “No,” or “Don’t know” (“Don’t know” was re-labeled as missing):

1. Did you ever cut the size of your meals or skip meals because you didn’t have resources (e.g., money to buy food; equipment to hunt, fish, or gather food; social connections to get food from; etc.) to get food?
2. Were you ever hungry but didn’t eat because you didn’t have resources to get food?
3. Did you ever not eat for a whole day because you didn’t have resources to get food?

### Statistical analysis

Descriptive characteristics of individuals by the proportion of individuals in each category of age, region, marital status, income, and the markers of food insecurity were calculated. Differences in sociodemographic characteristics were compared by sex using a chi-square test.

Dietary profiles were created using latent profile analysis (LPA), a latent variable mixture model, to identify the number of unobserved dietary profiles in the population (Bartholomew et al., 2011). This method creates mutually exclusive profiles, meaning that all individuals are assigned to a single profile each. A similar method, latent class analysis (LCA), has been used in identifying meal patterns in nutritional studies (O’Hara & Gibney, 2021). In contrast to LCA, LPA uses continuous/ordinal data for the creation of the profiles (Bartholomew et al., 2011), which is more in line with the data available from Q2017.

LPA was conducted using two datasets. The first included market and country food variables and the second included only country food variables (Online resource 1). These two analyses were conducted to identify the overall dietary profiles (based on market and country foods), and to further identify the types of country food consumption profiles in the population. Given the nature of dietary preferences, many of the distributions of variables were skewed. For example, some country foods were rarely consumed, and the distribution was highly skewed to the right. To avoid potential extreme cases (i.e., outliers) from influencing profile analysis, only foods consumed by at least 75% of the study population were included in the models, for a total of 53 food items. This provided a conservative cut-off to ensure that rarely consumed foods do not bias the estimation of dietary profiles by over-inflating their representation (or lack thereof). This led to the exclusion of the following dietary variables: pike/walleye, muskox, polar bear, walrus, Labrador tea, diet soft drinks, and energy drinks.

The best model was selected based on the optimal number of *k* unobserved profiles to describe the relationships between the variables (Spurk et al., 2020). To this end, fit performance scores, Akaike’s information criterion (AIC), Bayesian information criterion (BIC), and entropy values were compared. Lower values of AIC and BIC indicate better model fit. Entropy values range between 0 and 1, wherein values closer to 1 are considered to have better classification accuracy. Entropy levels above 0.80 indicate models with good fit. Each of the fit performance scores has its own set of disadvantages. For example, while the BIC and AIC can overestimate the number of underlying profiles, entropy can vary simply by the mere number of profiles rather than model fit (Spurk et al., 2020). Thus, in addition to a comparison of model fit performance scores, an inspection of the profile shapes in relation to their interpretability and individual profile sizes was also performed for final model selection.

After assigning each individual an overall dietary profile and a country food dietary profile based on the LPA model results, the dietary profile distributions by sociodemographic (age, sex, ecological region of residence) and responses to three food insecurity markers (skipping meals, being hungry, and not eating for a whole day, due to a lack of resources) variables were calculated. All distributions were weighted using survey weights calculated using sex, age, and ecological region distribution of the underlying Nunavik population in 2017 to ensure more representative population-level estimates (Hamel et al., 2020).

Multinomial logistic regression models were conducted to test adjusted associations between dietary profiles and sociodemographic and food insecurity variables. Odds ratios were calculated from the models. All models were adjusted for age and sex.

LPA models were conducted in R 3.6.1 using the tidyLPA package (Rosenberg et al., 2019). Weighted population distributions and multinomial logistic regression models were conducted in SAS 9.4 (SAS Institute Inc., Cary, NC).

## Results

After excluding individuals with missing data, 1176 individuals were included in the present study. The majority were aged 16–29 years (41.7–42.3%), followed by those aged 30–49 years and those ≥ 50 years (Table 1). More individuals were based in Hudson Bay, and almost half of the individuals had incomes less than $20,000 (even after excluding individuals aged below 20—data not shown). Males were more likely to report a personal income of $20,000–$59,999, whereas women were more likely to report higher personal incomes of ≥ $60,000. Likewise, 15.4% of women reported having at least some college versus 10.0% of men. Males also reported skipping meals, being hungry, and not eating all day due to a lack of resources significantly more often than females.

**Table 1:** Description of the population sociodemographic characteristics by sex

Characteristic	Females (%)	Males (%)	*p* value
Age			0.62
16–29 years	41.7	42.3	
30–49 years	33.7	34.2	
≥ 50 years	24.6	23.5	
Region			< 0.0001
Hudson Bay	41.5	43.9	
Hudson Strait	25.5	22.1	
Ungava Bay	33.0	34.1	
Marital status			0.88
Married or living with partner	46.7	47.2	
Single	53.2	52.8	
Missing	0.1	–	
Income			0.01
< $20,000	46.7	46.4	
$20,000–$59,999	23.7	34.2	
> $60,000	12.0	10.8	
Missing	17.7	8.6	
Education			0.04
< Grade 9/secondary 3	36.1	38.0	
Grade 9/secondary 3–high school diploma	46.2	52.0	
At least some college	15.4	10.0	
Missing	2.4	–	
Food insecurity markers			
Skipped or cut meals due to lack of resources	24.2	32.1	0.01
Missing	1.3	0.2	
Hungry but could not eat due to lack of resources	19.5	28.5	0.001
Missing	0.2	–	
Did not eat for a whole day due to lack of resources	10.2	18.4	0.0003
Missing	0.4	0.8	

Analyses used weights to account for sampling methodology and item non-response and thereby allow the results to be inferred to the target population; variance was estimated with the balanced repeated replication method. Differences by sex were calculated in complete datasets (i.e., they did not include “Missing” as a category)

**Fig. 2:**
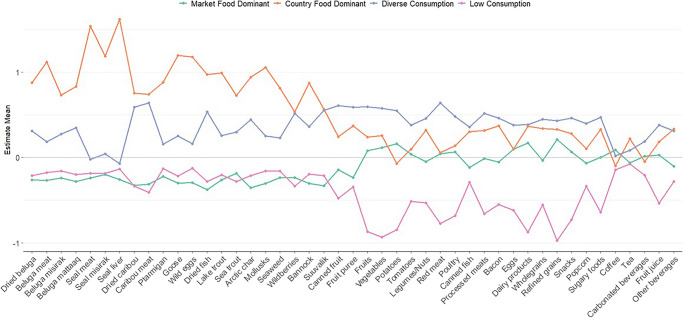
Dietary profile descriptions using country and market food variables. Sample mean represented by “zero” line

After running LPA using country and market food variables, the models with *k* = 4 profiles had the lowest AIC and BIC values (Table 2). Models with *k* = 5 profiles did not converge. In a further analysis of the profiles from the *k* = 4 model, differences by country versus market foods emerged (Fig. 2). The first dietary profile, *market food dominant* (depicted by the green line), included 42.0% of the study population and was characterized by a lower consumption frequency of country foods and a higher frequency of market foods. This profile is also defined by more frequent consumption of fruits, vegetables, poultry, dairy products, and refined grains (Online resource 2). The second dietary profile, *country food dominant* (depicted by the orange line), was the smallest group with 12.6% of the study population and was characterized by a higher consumption frequency of country foods and a lower consumption frequency of market foods. In comparison to the other profiles, this profile was particularly defined by consumption of beluga meat, seal meat, seal liver, wild birds, wild eggs, trout, mollusks, *bannock* (traditional hand-made bread), and tea (market). The market foods most frequently consumed in this profile included bacon, dairy, wholegrains, pureed fruits, and sugary foods. The third dietary profile, *diverse consumption* (depicted by the blue line), comprised 23.4% of the study population and included individuals who reported high consumption frequencies of both country and market foods. Caribou, *pitsik* (air-dried fish flesh, often made of Arctic char), and wild berries were among the mostly frequently consumed country foods, and red meat, canned fish, fruits, and vegetables were among the most frequently consumed market foods in the *diverse-consumption* profile. The fourth dietary profile, *low consumption* (depicted by the purple line), made up 21.9% of the study population, and included individuals who reported a low frequency of consumption of both market and country foods. However, this group also appeared to consume country foods more frequently than market foods, particularly ptarmigan, wild bird eggs, seal liver, mollusks, and seaweed. Their diet also included frequent consumption of carbonated beverages, canned fish, canned fruits, and pureed fruits, and they were the least likely to consume vegetables and dairy products compared to other dietary profiles.

**Table 2:** LPA model fit statistics (AIC, BIC, and entropy) across *k* = 2 to *k* = 5 profiles

Number of profiles	AIC	BIC	Entropy
Models with market and country food variables (overall dietary profiles)			
2	146094.9	146784.5	0.88
3	144678.9	145601.7	0.86
4	143896.3	145052.5	0.86
5	Did not converge	Did not converge	Did not converge
Models with country food variables only (country food dietary profiles)			
2	672265.9	675511.1	0.86
3	663843.0	668203.8	0.83
4	660232.3	665708.6	0.80
5	655883.6	662475.6	0.83

**Fig. 3:**
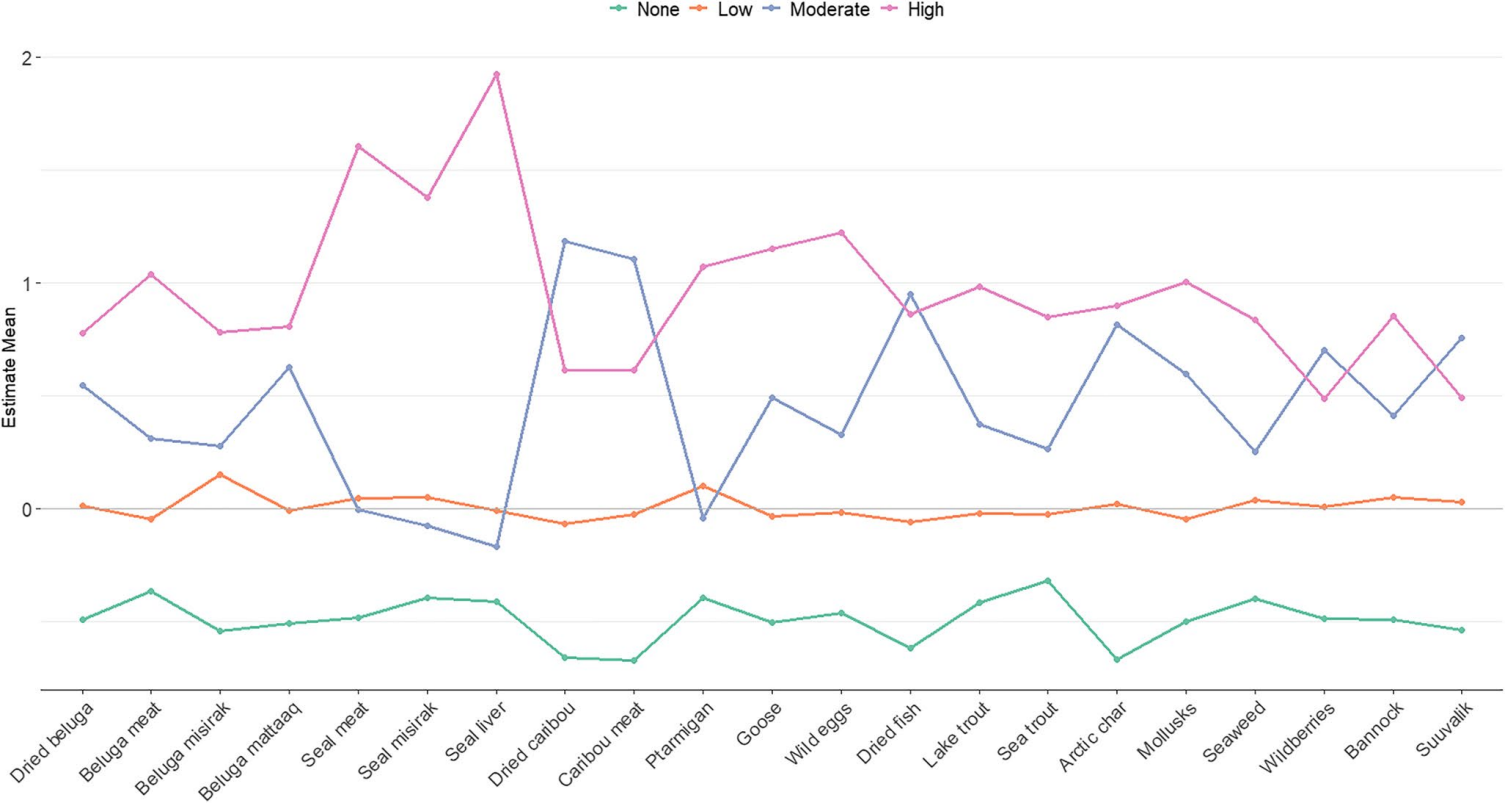
Dietary profile descriptions using only country variables. Sample mean represented by “zero” line

LPA models incorporating only country food variables had the lowest AIC and BIC with *k* = 5 profiles (Table 2). However, the interpretation of these dietary profiles was not clear, and the model included profiles with small sample sizes of less than 50 individuals (Online resource 4). As such, the model with *k* = 4 profiles was selected for further analysis (Fig. 3). The country food dietary profiles depicted differences in the frequency of consumption, such that the first profile indicated no or very little consumption of country foods (*none*, depicted by the green line) and included 31.5% of the study population, followed by *low* frequency of consumption (depicted by the orange line) including 42.1% of the study population, *moderate* frequency of consumption (depicted by the blue line) including 15.6% of the study population, and finally, *high* frequency of consumption of country foods (depicted by the purple line) including 10.8% of the study population. There were also differences in the types of country foods consumed between the *moderate* and *high* profiles, similar to the differences observed between the *country food–dominant* and *diverse-consumption* groups observed in the previous model. The *moderate* dietary profile was primarily characterized by a higher consumption frequency of beluga *nikku* (air-dried meat), beluga *mattaaq* (skin and blubber), caribou, *pitsik* (air-dried fish flesh), Arctic char (frozen, raw, cooked), wild berries, and *suuvalik* (traditional dish made of fish roe, seal blubber or Crisco oil, and wild berries). By contrast, the *high* dietary profile was more diverse and characterized by a higher consumption frequency of beluga meat, seal meat, seal *misirak* (rendered and aged fat), wild birds (ptarmigan, goose), wild eggs, diverse seafood (Arctic char, lake trout, sea trout, mollusks, and seaweed), and *bannock*.

For the overall dietary profiles, the *market food–dominant* (53.1%), *diverse-consumption* (52.8%), and *low*-*consumption* (52.8%) profiles comprised a greater proportion of women than men, whereas women represented only 26.7% of individuals in the *country food–dominant* profile (Table 3). Compared to other dietary profiles, Nunavimmiut in the *market food–dominant* group were more likely to be aged 30–49 years (39.4%), while those in the *country food–dominant* group were more likely to be aged 16–29 years (51.2%) or ≥ 50 years (28.1%), and Nunavimmiut in the *diverse-consumption* group were also more likely to be aged 16–29 years (47.2%). Trends by ecological region were less clear. Finally, the *low-consumption* group had the highest proportion of Nunavimmiut reporting being hungry (30.0%) or not eating a whole day (20.9%) due to a lack of resources.

**Table 3:** Distribution of overall dietary profiles by sex, age, region, and food insecurity markers in Nunavik

	Market food dominant (42.0%)	Country food dominant (12.6%)	Diverse consumption (23.4%)	Low consumption (21.9%)
Sex (%)				
Female	53.1	26.7	52.8	52.8
Male	46.9	73.3	47.2	47.2
Age (%)				
16–29 years	37.6	51.2	47.2	40.5
30–49 years	39.4	20.7	29.8	36.5
≥ 50 years	23.0	28.1	23.0	23.1
Region (%)				
Hudson Strait	20.8	27.7	25.3	27.1
Hudson Bay	41.1	51.6	35.4	45.4
Ungava Bay	38.0	20.7	39.3	27.4
Food insecurity markers (%)				
Skipped or cut meals due to lack of resources	24.7	43.0	22.9	32.8
Hungry but could not eat due to lack of resources	20.9	24.7	22.3	30.0
Did not eat for a whole day due to lack of resources	13.1	13.7	10.5	20.9

Results from adjusted multinomial logistic regression models mirrored the results in Table 4. Women were less likely than men to be in the *country food–dominant* (OR 0.33, 95% CI 0.20–0.52) profile versus the *market food–dominant* profile. Likewise, Nunavimmiut aged 30–49 years were also less likely than those aged 16–29 years to be in the *country food–dominant* (OR 0.38, 95% CI 0.21–0.70) and *diverse-consumption* (OR 0.60, 95% CI 0.41–0.88) profiles versus the *market food–dominant* profile. Nunavimmiut living in the Hudson Strait or Hudson Bay versus Ungava Bay were more likely to be in the *country food–dominant* (OR 2.75, 95% CI 1.51–4.99; OR 0.37, 95% CI 1.39–4.04) and *low-consumption* (OR 1.94, 95% CI 1.22–3.05; OR 1.54, 95% CI 1.01–2.35) groups, compared to the *market food–dominant* profile. Finally, compared to those in the *market food–dominant* profile, Nunavimmiut in the *low-consumption* profile were more likely to report skipping meals (OR 1.64, 95% CI 1.12–2.40), being hungry but unable to eat (OR 1.67, 95% CI 1.10–2.52), and not eating for a whole day (OR 1.78, 95% CI 1.08–2.94). Nunavimmiut in the *country food–dominant* profile were also more likely to report skipping meals (OR 2.19, 95% CI 1.33–3.60) compared to those in the *market food–dominant* profile.

**Table 4:** Odds ratios and associated 95% confidence intervals from adjusted multinomial logistic regression models comparing overall dietary profiles and sociodemographic variables

	Country food dominant vs. Market food dominant	Diverse consumption vs. Market food dominant	Low consumption vs. Market food dominant
	OR (95% CI)	OR (95% CI)	OR (95% CI)
Sex			
Female (reference: male)	0.33 (0.20–0.52)**	0.96 (0.68–1.37)	0.93 (0.65–1.33)
Age			
16–29 years	1.00	1.00	1.00
30–49 years	0.38 (0.21–0.70)**	0.60 (0.41–0.88)**	0.89 (0.59–1.35)
≥ 50 years	0.90 (0.51–1.58)	0.80 (0.52–1.25)	0.88 (0.59–1.33)
Region			
Hudson Strait	2.75 (1.51–4.99)**	1.20 (0.78–1.84)	1.94 (1.22–3.05)**
Hudson Bay	2.37 (1.39–4.04)**	0.86 (0.56–1.31)	1.54 (1.01–2.35)**
Ungava Bay	1.00	1.00	1.00
Food insecurity markers			
Skipped or cut meals due to lack of resources (reference: no)	2.19 (1.33–3.60)**	0.96 (0.64–1.43)	1.64 (1.12–2.40)**
Hungry but could not eat due to lack of resources (reference: no)	1.10 (0.61–1.99)	1.07 (0.71–1.61)	1.67 (1.10–2.52)**
Did not eat for a whole day due to lack of resources (reference: no)	0.81 (0.39–1.67)	0.73 (0.41–1.32)	1.78 (1.08–2.94)**

All models adjusted for age and sex. *N* for all models = 1139

**p < 0.05

For country food dietary profile, the *none* and *low* country food dietary profiles had higher proportions of women (53.4%; 53.7%) and those aged 30–49 years (37.1%; 39.3%) compared to the *moderate* and *high* profiles, whereas the *moderate* and *high* profiles had higher proportions of men (50.2%; 77.7%) and those aged 16–29 (56.1%; 47.4%) and ≥ 50 (22.7%, 30.0%) years (Table 5). While individuals in the *high*-consumption group reported skipping meals the most (46.2%), individuals in the *low*-consumption group had the highest proportion of individuals not eating for a whole day due to a lack of resources (14.7%).

**Table 5:** Distribution of country food dietary profiles by sex, age, region, and food insecurity markers in Nunavik

	None (31.5%)	Low (42.1%)	Moderate (15.6%)	High (10.8%)
Sex (%)				
F	53.4	53.7	49.8	22.3
M	46.6	46.3	50.2	77.7
Age (%)				
16–29 years	37.9	38.4	56.1	47.4
30–49 years	37.1	39.3	21.3	22.6
≥ 50 years	25.0	22.3	22.7	30.0
Region (%)				
Hudson Strait	17.3	27.0	25.2	27.8
Hudson Bay	48.3	36.3	44.8	47.8
Ungava Bay	34.3	36.6	30.0	24.4
Food insecurity markers (%)				
Skipped or cut meals due to lack of resources	24.4	28.6	23.3	46.2
Hungry but could not eat due to lack of resources	23.9	24.6	21.8	26.1
Did not eat for a whole day due to lack of resources	14.3	16.9	7.8	14.7

Adjusted multinomial logistic regression results showed that women were less likely than men to be in the *high* versus *none* country food dietary profile (OR 0.26, 95% CI 0.16–0.43), and Nunavimmiut aged 30–49 years versus Nunavimmiut aged 16–29 years were less likely to be in the *moderate* (OR 0.39, 95% CI 0.23–0.65) or *high* (OR 0.46, 95% CI 0.24–0.88) versus *none* country food profiles (Table 6). Individuals living in the Hudson Strait versus Ungava Bay were more likely to report any consumption of country food, with linear increases from *low* (OR 1.50, 95% CI 1.00–2.24) to *moderate* (OR 1.70, 95% CI 1.01–2.86) and *high* (OR 2.58, 95% CI 1.33–4.99). Individuals in the *high* country food profile were more likely to report skipping meals (OR 2.33, 95% CI 1.36–4.01) versus *none*, whereas individuals in the *moderate* country food profile were less likely to report not eating for a whole day (OR 0.45, 95% CI 0.21–0.96) compared to *none*.

**Table 6:** Odds ratios and associated 95% confidence intervals from adjusted multinomial logistic regression models comparing country food dietary profiles and sociodemographic variables

	Low vs. None	Moderate vs. None	High vs. None
	OR (95% CI)	OR (95% CI)	OR (95% CI)
Sex			
Female (reference: male)	1.05 (0.78–1.43)	0.86 (0.56–1.30)	0.26 (0.16–0.43)**
Age			
16–29 years	1.00	1.00	1.00
30–49 years	1.00 (0.70–1.42)	0.39 (0.23–0.65)**	0.46 (0.24–0.88)**
≥ 50 years	0.80 (0.55–1.16)	0.61 (0.37–1.03)*	0.90 (0.49–1.66)
Region			
Hudson Strait	1.50 (1.00–2.24)**	1.70 (1.01–2.86)**	2.58 (1.33–4.99)**
Hudson Bay	0.72 (0.50–1.03)*	1.06 (0.66–1.68)	1.47 (0.84–2.56)
Ungava Bay	1.00	1.00	1.00
Food insecurity markers			
Skipped or cut meals due to lack of resources (reference: no)	1.21 (0.85–1.73)	0.93 (0.57–1.50)	2.33 (1.36–4.01)**
Hungry but could not eat due to lack of resources (reference: no)	1.03 (0.73–1.46)	0.80 (0.48–1.35)	0.94 (0.51–1.72)
Did not eat for a whole day due to lack of resources (reference: no)	1.22 (0.77–1.93)	0.45 (0.21–0.96)**	0.75 (0.35–1.62)

All models adjusted for age and sex. *N* for all models = 1139

*0.05 ≤ *p* ≤ 0.10; **p < 0.05

## Discussion

In the first known study of its kind in the region, we identified four overall dietary profiles based on market and country foods, and four country food dietary profiles among Nunavimmiut in 2017. Overall, women and Inuit adults aged 30–49 years were more likely to have a market food–dominant dietary profile. Men, younger Inuit (aged 16–29 years), and older Inuit (≥ 50 years) had a higher likelihood of frequent consumption of country foods. Younger Inuit were also more likely to belong to the moderate country food dietary profile compared to other age groups. Inuit living in the Hudson Strait reported higher consumption of country foods compared to those living in other regions, particularly Ungava Bay. Individuals in the low-consumption profile also reported high food insecurity across all three markers.

There are complex dynamics between culture, colonialism, generations, wage-based employment, and availability of resources for harvesting that influence country food harvest and sharing, dietary patterns, and the purchase of market foods (Little et al., 2021; Todd, 2010). The enduring impacts of colonialism, in particular, have been key in the increased representation of market foods in contemporary diets (Hackett et al., 2016; Hoover et al., 2016). Residential schools in the Canadian Arctic have a unique history because they were implemented much later compared to residential schools in the south. Most children did not attend residential schools in northern communities until well into the 1950s, and the majority of students only attended residential schools after 1964 until the demise of these schools in the late 1990s (*Residential Schools- Inuit Experience*, 2019; Truth and Reconciliation Commission of Canada, 2016). As such, many residential school survivors are alive today in the north, and the majority of them would have been in the age range of 23–58 years in 2017 (assuming children began participation in the system—often involuntarily or through coercion—at the age of 5 years). Our results showed that Inuit aged 30–49 years were most likely to eat predominantly market foods and least likely to rely heavily on country foods in their diet, and this may be explained, among other factors, by the lasting effects of residential schools, which systematically and intentionally disrupted the intergenerational traditional knowledge transmission and livelihoods of Inuit families. Residential schools additionally contributed to the transformation of the region’s traditional-based lifestyles and economies due to the long distances children had to travel in order to go to school (Truth and Reconciliation Commission of Canada, 2016). Parents would often opt to settle in nearby communities on a year-round basis to live close to their children, thus reducing the ability of parents to sustain their traditional lifestyles. Additionally, many children attending residential schools were denied opportunities to participate in land-based subsistence activities, and survivors adopted (or were forced to adopt) lifestyles similar to colonial settler Canadians as an aftermath of the doctrine of assimilation (Inuit Tapiriit Kanatami, 2017). These events also coincided with a number of key events that impacted the ability to hunt or fish on the land or pass down generational knowledge, including the forced relocation of whole Inuit communities to unknown territories, the over-hunting of animals by colonial commercial hunting practices, restrictions placed on the Inuit on the type and number of animals that could be harvested, and the culling of Inuit sled dogs by colonial police enforcement (Inuit Tapiriit Kanatami, 2017). Additionally, Inuit aged 30–49 years are less likely to own a home and have less access to hunting resources, which can be costly to purchase and maintain, further impacting their consumption of country foods (NRBHSS, personal communication, February 1, 2022).

Despite the historic and current challenges, consumption of country foods is an integral part of contemporary Inuit culture (Lamalice et al., 2020), and the majority of Inuit regularly consume at least some country foods (68.5% reported low to high consumption of country foods in this population survey). There has been a resurgence in interests surrounding country food consumption due to their important cultural connections, superior nutritional quality, and potential to contribute to community and household food security (Breton-Honeyman et al., 2021; Duhaime et al., 2004; Huet et al., 2012; Inuit Tapiriit Kanatami, 2017; ITK, 2021; Kenny et al., 2018; Lawn & Harvey, 2003; Little et al., 2021; NIEDB, 2019). In fact, previous research has shown that Inuit households reporting having time and resources to harvest (i.e., money, equipment, etc.), as well as those with a hunter in the home, are less likely to be food insecure (Huet et al., 2012). In Nunavik, as across Inuit Nunangat, community initiatives, such as family houses (safe spaces for families in crisis), are running local food programs aimed at increasing access to foods, including country foods. The Sirivik Food Centre in Inukjuak and the distribution of food to pregnant women through the Ilagiilluta program’s activities are among those initiatives (Nunavik Regional Board of Health and Social Services, 2022a; Sirivik, 2021). The Nunami program was developed by the NRBHSS to promote mental health and wellness, Inuit cultural identity, and healthy nutrition, and also provides funding to communities and organizations for land-based activities (Nunavik Regional Board of Health and Social Services, 2022b). Inuktitut language, Nunavimmiut culture and land survival, and cooking classes are also taught to the youth in Nunavik schools (Kativik Ilisarniliriniq, 2022). Additionally, the Hunter Support Program (HSP), created under the James Bay and Northern Quebec Agreement and coordinated by the Kativik Regional Government, is key in enabling country food access (Kativik Regional Government, 2018). The program’s objective is to “favour, encourage, and perpetuate the hunting, fishing and trapping activities of the Inuit as a way of life, and to guarantee the Inuit communities a supply of produce from such activities” (Québec, 2021). Community freezers and financial support for hunting equipment are managed locally as part of the HSP in all Nunavik communities.

Such programs, which promote access, affordability, sharing, and consumption of country foods in Nunavik (with many aimed at young Inuit), could, in part, explain the more frequent consumption of country foods among younger Inuit aged 16–29 years. Moreover, Inuit grandparents play a major role in the upbringing of their grandchildren (Briggs, 2017; Milan et al., 2015). Thus, contrary to Inuit aged 30–49 years old, younger Inuit aged 16–29 years may have been more exposed to country foods throughout their upbringing with their grandparents. Previous studies in other Indigenous communities found that youth describe traditional foods as a way to connect inter-generationally (Hanemaayer et al., 2022) and would consume traditional foods more regularly if they were available in their homes (Hlimi et al., 2012). While young Inuit made up a large proportion of individuals in the *country food–dominant* profile, in contrast to the oldest group (50+ years), young Inuit were slightly more likely to eat a diverse diet consisting of a high consumption frequency of both country and market foods. This may be a result of the influence of open communication with other regions of the world via increased transportation and the Internet (Worden et al., 2020). It is also interesting to note that the types of country foods more likely to be consumed by younger Inuit also varied in comparison to those consumed by older Inuit. Specifically, young Inuit were more likely to frequently consume beluga *mattaaq*, caribou, Arctic char, and berries. Caribou meat and beluga *mattaaq*, for example, are increasingly viewed as a delicacy in some regions of Nunavik (NRBHSS, personal communication, February 1, 2022). This contrasts with the more frequent consumption of marine mammal meats and a generally more diverse diet of country foods (including a variety of species and consumption of more animal parts) among older Inuit. There has been messaging in Nunavik regarding the high concentrations of environmental contaminants in some parts of marine mammals (e.g., beluga meat and *nikku*) which may have indirectly led to overall reduced consumption of marine mammal foods; however, the impact of this messaging on dietary choices is unclear with some evidence suggesting that target audiences did not register or act on the messaging provided (Boyd et al., 2019; Krümmel & Gilman, 2016; Little et al., 2021; Mead et al., 2010). Future studies should attempt to determine the influence of all these interventions (cultural, food insecurity–based, environmental chemicals in foods) on country food consumption among youth. Also, more detailed analysis of diet in relation to community food program use may be warranted.

While all genders participate actively in country food harvest and preparation, men are traditionally the hunters in Inuit culture; thus, households without a hunter, including single mothers, tend to rely more heavily on market foods and may experience more difficulties accessing country foods (Duhaime et al., 2004; Inuit Tapiriit Kanatami, 2017). This may partially explain our results identifying women as more likely to eat predominantly market foods. Additionally, women in Q2017 had higher incomes and levels of education as compared to men. This is consistent with other studies conducted in Nunavut that reported women are more likely to participate in the labour force compared to men (Quintal-Marineau, 2017a), playing an important role for monetary contributions of subsistence practices (e.g., hunting) (Quintal-Marineau, 2017b; Quintal-Marineau & Wenzel, 2019). As higher income and education are associated with more economically driven occupations, it is likely that women have less time to harvest and prepare country foods, increasing their reliance on market foods. However, the large differences in the frequency of country food consumption in males versus females remain to be further investigated.

The *low-consumption* profile was associated with skipping meals, being hungry, and not eating for a whole day due to a lack of resources. Food security is a major problem in Nunavik (Furgal et al., 2022); the 2017 Aboriginal Peoples Survey reported that 77% of Inuit aged 15 years and up in Nunavik were food insecure (ITK, 2021). Nevertheless, the *low-consumption* dietary profile had more country food consumption compared to market food consumption, especially certain country foods like ptarmigan, wild bird eggs, mollusks, and seaweed. Ptarmigan is usually hunted using small-caliber rifles (as opposed to shotguns) (Kwan, 2018), thus requiring less equipment than hunting marine mammals and caribou. Furthermore, the HSP supplies thousands of ptarmigans to community freezers to be freely available to anyone each year (Kwan, n.d.). Wild bird egg, mollusk, and seaweed harvest is also accessible without specialized hunting equipment. Mussels and seaweed can be harvested almost year-round by foot at low tide, particularly in Ungava Bay communities which are known to have some of the highest tides in Canada (Rapinski et al., 2018). The market foods consumed by individuals in the *low-consumption* profile also included calorie-dense foods that require less preparation, such as canned fish, sugary foods, and carbonated beverages. The low consumption of fruits, vegetables, and dairy products among those in the *low-consumption* profile was consistent with another study conducted in the Inuvialuit Settlement Region, Nunavut, and Nunatsiavut that also reported low consumption of these foods among food-insecure Inuit (Huet et al., 2012). Nunavimmiut in the *country food–dominant* group also reported a higher rate of skipping meals; however, this may not be indicative of challenges to food access, given that Inuit participating in traditional lifestyles are more likely to eat throughout the day rather than have distinct meals (Huet et al., 2012), and are likely to feel fuller after a country food meal. They are also more dependent on the success of the hunt and the availability of wildlife in the surrounding area, which may be compounded by climate change (Little et al., 2021).

The distribution of the dietary profiles by region was as expected. Inuit living in Hudson Strait and Hudson Bay were more likely to have a *country food–dominant* profile compared to individuals in Ungava Bay. Ungava Bay includes Kuujjuaq, the largest village in Nunavik and the capital of the Kativik Regional Government. Inuit in Kuujjuaq have higher access to market foods compared to those in other regions and are less likely to consume country foods predominantly. The Leaf River caribou herd also migrates through the Hudson Bay and Hudson Strait region, and this herd is more abundant than the George River and Torngat Mountain caribou herds found in Ungava Bay (Committee on the Status of Endangered Wildlife in Canada (COSEWIC), 2018). Inuit in Hudson Strait were also more likely to have a *high* country food profile including increased frequency of beluga consumption. Beluga is largely hunted from the Hudson Strait in Nunavik, making beluga products more accessible in this region (DFO, 2018).

This study identified dietary profiles in a representative population with a large sample size. However, there were some limitations to the study. The food frequency questionnaire only provided information on the frequency of consumption of a type of food but did not provide information on the *quantity* consumed by study individuals. Study individuals provided consumption frequencies related to the previous 3 months, and consumption of country foods is highly variable depending on the season and the year (Kuhnlein et al., 1996; de Moraes Pontual, 2021; Wesche & Chan, 2010). The study was conducted from August to October, minimizing variability due to seasonality in the responses across individuals, but this may still have introduced some bias. Similarly, Inuit partners raised the issue that caribou was not accessible to all in 2017 compared to previous and more recent years. Additionally, in contrast to the food frequency questionnaire, the food insecurity questions were asked with regard to the year prior to the survey. Missingness related to some variables (such as income) led to a smaller subsample size, but analyses comparing the age, sex, and regional distribution of participants with missing socioeconomic and/or dietary diet and the subsample revealed no differences in the distribution (data not shown). Due to the length of the questionnaire, there may have been some respondent fatigue. Unmeasured residual confounding may have biased the results of the multinomial logistic regression, including community size, hunting quotas, and availability of a food item from one community to another due to season and regional food preferences (Guo et al., 2015). Some community food cooperatives may also lack human capital for continued access to market and country foods (NRBHSS, personal communication, February 1, 2022). Complementary qualitative studies will help add to the nuanced understanding of dietary profiles and food preferences by age, sex, and community. Finally, while the survey weights allow for generalizing results to the Nunavik population, the modest participation rate may have reduced some of that generalizability.

## Conclusion

This study aimed to identify dietary patterns in youth and adult Inuit living in Nunavik. Four overall dietary profiles and four country food dietary profiles were identified. We observed more frequent consumption of country foods among males, older Inuit (aged 50+ years), and Inuit living in the Hudson Strait. Alternatively, females and Inuit aged 30–49 years reported more frequent consumption of market foods. There was also evidence of younger Inuit aged 16–29 years consuming country foods more frequently compared to those aged 30–49 years. Individuals in the market food–dominant group more frequently consumed healthier store-bought options compared to those in the diverse- and low-consumption groups. These profiles will be used for further understanding of underlying sociocultural determinants of dietary patterns in Nunavik subpopulations, for further study of nutritional status, contaminant exposure, and physical and mental health outcomes, and to provide context for future nutritional programs.

## Contributions to knowledge

What does this study add to existing knowledge?

- The dietary profiles provided a global picture of dietary consumption in Nunavik subpopulations.
- While almost half of the Nunavik population consume predominantly market foods, over a fifth of the population continue to consume predominantly country foods.
- The differences across profiles are in line with cultural expectations, historical colonial influences, and subsequent country food promotion programs for Inuit youth.
- Inuit aged 50+ and Inuit aged 16-29 years were more likely to have higher country food consumption patterns compared to Inuit aged 30-49 years. Women and Inuit living in Ungava Bay were more likely to have patterns with higher market food consumption.

What are the key implications for public health interventions, practice or policy?

- These profiles can be used for further study of dietary patterns on nutritional status, contaminant exposure, and physical and mental health.
- Information on dietary patterns can be used to provide context for future nutritional and health programs.

### Supplementary Information

ESM 1 (DOCX 78 kb)

## Data Availability

The datasets generated and/or analyzed during the current study are not publicly available due to the sensitive nature of the data.
