# Supplementary material for: Sociodemographic patterning of dietary profiles among Inuit youth and adults in Nunavik, Canada: a cross-sectional study
Source: Can J Public Health. 2022 Dec 8;115(Suppl 1):66–82. doi: 10.17269/s41997-022-00724-7 (PMC10830983; doi:10.17269/s41997-022-00724-7)
Supplement: Supplementary file 1 — (DOCX 78 kb) [file 41997_2022_724_MOESM1_ESM.docx]

**Supplementary material for “Socio-demographic patterning of dietary profiles among adults and young Inuit in Nunavik, Canada: A cross-sectional study” in the Canadian Journal of Public Health**

Amira Aker^1,2^, Pierre Ayotte^1-3^, Chris Furgal^4^, Tiff-Annie Kenny^2^, Matthew Little^5^, Marie-Josée Gauthier^6^, Amélie Bouchard^6^, Mélanie Lemire^1,2,7^

1 Axe santé des populations et pratiques optimales en santé, Centre de recherche du CHU de Québec-Université Laval, Québec, Quebec, Canada

2 Département de médecine sociale et préventive, Université Laval, Québec, Quebec, Canada

3 Centre de Toxicologie du Québec, Institut National de Santé Publique du Québec, Québec, Canada

4 Department of Indigenous Studies, Trent University, Peterborough, Onatrio, Canada

5 School of Public Health and Social Policy, University of Victoria, Victoria, Canada

6 Nunavik Regional Board of Health and Social Services, Kuujjuaq, Quebec, Canada

7 Institut de biologie intégrative et des systèmes (IBIS), Université Laval, Quebec, Quebec, Canada

Corresponding author: Mélanie Lemire; [melanie.lemire@crchudequebec.ulaval.ca](mailto:melanie.lemire@crchudequebec.ulaval.ca); +1 418-525-4444 x 81967

Online Resource 1: List of foods included in the creation of the dietary profiles

| **Food item** | **Description (if any)** | **Frequency distribution of categories** |
| --- | --- | --- |
| **Market foods** |  |  |
| Fruits | Orange, banana, apple, pear, store-bought berries, etc. (fresh or frozen) | 1 3.7%  2 16.1%  3 21.7%  4 31.4%  5 27.1% |
| Vegetables | Green, leafy vegetables (iceberg, romaine or leaf lettuce, spinach), carrots, broccoli, cauliflower, cabbage, pepper (green, red, yellow), onions, corn, cucumber, celery, mushrooms, mixed vegetables (fresh, frozen, canned) | 1 4.8%  2 20.3%  3 25.0%  4 29.8%  5 20.2% |
| Canned fruits | - | 1 33.7%  2 25.9%  3 20.4%  4 10.6%  5 9.3% |
| Fruit puree | - | 1 60.8%  2 16.9%  3 10.6%  4 5.6%  5 6.2% |
| Potatoes | Mashed, baked, or boiled | 1 7.9%  2 19.8%  3 30.4%  4 30.9%  5 11.0% |
| Tomatoes | Whole or canned, or V8 juice | 1 39.0%  2 22.7%  3 19.3%  4 13.3%  5 5.7% |
| Legumes and nuts | Beans, lentils, chickpeas (baked, canned), peanut butter, nuts (almonds, etc.), peanuts, sunflower seeds | 1 28.9%  2 30.6%  3 17.5%  4 11.9%  5 11.0% |
| Red meat | Beef or pork as main dish (steak, roast, chops, etc.), hamburger (lean or regular) | 1 6.4%  2 30.6%  3 33.1%  4 24.1%  5 5.7% |
| Poultry | Chicken/turkey (breast, legs) | 1 8.7%  2 32.2%  3 31.6%  4 22.8%  5 4.9% |
| Canned fish | Salmon, sardines, tuna | 1 64.8%  2 22.4%  3 9.5%  4 2.0%  5 1.3% |
| Processed meat | Sliced or processed meat (ham, salami, bologna, Kam/Spam, etc.), sausage (small links or canned), hot dogs, beef jerky (dried, canned, stewed or corned), chicken nuggets, chicken wings, fried chicken | 1 3.2%  2 26.4%  3 29.7%  4 27.2%  5 13.5% |
| Bacon |  | 1 17.3%  2 25.3%  3 22.5%  4 17.8%  5 17.1% |
| Eggs | Chicken eggs | 1 11.1%  2 13.2%  3 18.0%  4 26.5%  5 31.2% |
| Dairy | Milk, chocolate milk, yoghurt (container or drink), cheese (plain or as part of a dish including cheddar, mozzarella), processed cheese (Kraft Singles or Cheez Whiz) | 1 2.5%  2 9.8%  3 13.3%  4 29.1%  5 45.3% |
| Whole grain | Whole wheat bread or other whole grains, hot cereals (oatmeal, etc.) | 1 33.5%  2 16.1%  3 16.0%  4 14.9%  5 19.5% |
| Refined grains | White bread, cold cereals (cornflakes, special K, etc.), pasta, rice | 1 0.1%  2 4.0%  3 10.2%  4 30.7%  5 54.9% |
| Snacks | Noodle soup, crackers, pizza, potato chips, corn tortilla chips, french fries or poutine | 1 0.3%  2 9.0%  3 18.2%  4 34.7%  5 37.8% |
| Popcorn | - | 1 55.7%  2 25.6%  3 10.7%  4 4.4%  5 3.7% |
| Sweets | Cookies, cakes, muffins (homemade or ready made), chocolates (Hershey's, Aero, M&M's, etc.) or candy bars (Snickers, Reeses, Mars, etc.) (bar or pack), candies (gummies, jelly, etc.), chocolate spreads (Nutella), jam (homemade or ready made), marmalade, ice cream | 1 2.9%  2 17.6%  3 23.1%  4 27.9%  5 28.4% |
| Coffee | - | 1 20.4%  2 4.0%  3 4.4%  4 7.0%  5 64.2% |
| Tea | - | 1 30.1%  2 12.6%  3 11.7%  4 9.5%  5 36.1% |
| Traditional tea | Labrador tea (mamaittuqutik) | 1 78.5%  2 9.2%  3 5.1%  4 3.0%  5 4.3% |
| Carbonated beverages - regular | Soft drinks (regular only) | 1 23.8%  2 8.6%  3 10.0%  4 17.2%  5 40.4% |
| Carbonated beverages - diet | Soft drinks (diet, sugar-free, or low-calorie) | 1 83.9%  2 3.7%  3 2.9%  4 3.4%  5 6.1% |
| Carbonated beverages - energy | Energy drinks (Red Bull, Monster, etc.) | 1 89.5%  2 4.6%  3 1.8%  4 1.1%  5 3.1% |
| Fruit juices | Real fruit juices (100% pure), bottled or canned, frozen concentrate and diluted | 1 14.7%  2 13.6%  3 15.6%  4 23.1%  5 34.0% |
| Other beverages | Fruit cocktail (Punch, Sunny D), powdered drinks (Kool-Aid), sport drinks (Gatorade, Powerade) | 1 41.5%  2 16.8%  3 12.1%  4 13.5%  5 16.2% |
| **Country foods** |  |  |
| Beluga nikku | Dried beluga meat | 1 47.6%  2 34.9%  3 11.6%  4 1.7%  5 4.3% |
| Beluga meat | Fresh, cooked, frozen | 1 62.9%  2 23.5%  3 6.5%  4 4.8%  5 2.3% |
| Beluga misirak/ursuk | Misirak: Rendered beluga blubber  Ursuk: Rendered and aged beluga blubber | 1 40.9%  2 26.9%  3 13.0%  4 12.4%  5 6.8% |
| Beluga mattaaq | Beluga skin and underlying fatty layer | 1 29.1%  2 49.6%  3 10.2%  4 7.8%  5 3.3% |
| Seal meat | Fresh, cooked, frozen | 1 55.3%  2 32.9%  3 7.2%  4 2.6%  5 2.0% |
| Seal misirak/ursuk | Misirak: Rendered seal blubber  Ursuk: Rendered and aged seal blubber | 1 68.4%  2 18.4%  3 6.3%  4 4.8%  5 2.2% |
| Seal liver | - | 1 69.3%  2 23.2%  3 5.0%  4 1.4%  5 1.1% |
| Walrus meat | - | 1 91.9%  2 6.8%  3 0.6%  4 0.6%  5 0.1% |
| Caribou nikku | Dried caribou meat | 1 10.8%  2 43.4%  3 19.4%  4 18.3%  5 8.0% |
| Caribou meat | - | 1 8.3%  2 40.2%  3 20.5%  4 22.3%  5 8.6% |
| Muskox meat | - | 1 96.7%  2 2.6%  3 0.1%  4 0.4%  5 0.1% |
| Polar bear meat | - | 1 97.4%  2 2.1%  3 0.05%  4 0.1%  5 0.3% |
| Ptarmigan/partridge | - | 1 60.8%  2 29.3%  3 5.5%  4 3.1%  5 1.4% |
| Goose | Canada goose or white goose | 1 30.2%  2 46.7%  3 12.0%  4 8.1%  5 2.9% |
| Wild bird eggs | Duck, geese, murre/seagulls | 1 58.5%  2 31.2%  3 5.6%  4 2.9%  5 1.7% |
| Dried fish | Mostly made of Arctic char | 1 25.2%  2 45.1%  3 15.2%  4 10.8%  5 3.6% |
| Lake trout | Fresh, cooked, or frozen, not dried | 1 49.2%  2 35.2%  3 9.5%  4 4.3%  5 1.7% |
| Brook, sea trout, or salmon | Fresh, cooked or frozen, not dried | 1 61.9%  2 27.9%  3 6.4%  4 2.9%  5 0.9% |
| Arctic char | Fresh, cooked or frozen, not dried | 1 17.4%  2 42.6%  3 18.3%  4 16.4%  5 5.3% |
| Pike or walleye | - | 1 94.2%  2 4.1%  3 0.9%  4 0.2%  5 0.7% |
| Other fish | Lake whitefish (coregone) or sculpin (ugly fish) | 1 75.3%  2 17.2%  3 4.9%  4 1.3%  5 1.4% |
| Mollusks | Mussels, scallops, clams, and urchins | 1 41.4%  2 43.2%  3 9.0%  4 5.0%  5 1.4% |
| Seaweed | - | 1 73.3%  2 19.6%  3 3.9%  4 2.1%  5 1.1% |
| Wild berries | Cloudberries, blackberries, blueberries, redberries, cranberries | 1 12.4%  2 35.3%  3 21.2%  4 18.1%  5 13.1% |
| Bannock | Flat quick bread | 1 16.6%  2 30.8%  3 20.4%  4 12.8%  5 19.3% |
| Suuvalik/uarutilik | Suuvalik: fish eggs, blackberries or blueberries, and fat; Uarutilik: cooked fish, blackberries or blueberries, and fat | 1 30.1%  2 31.8%  3 17.8%  4 13.2%  5 7.0% |
| 1: Never or less than once a month  2: 1-3 times a month  3: Once a week  4: 2-6 times a week  5: Daily  Analyses used weights to account for sampling methodology and item non-response and thereby allow the results to be inferred to the target population; variance was estimated with the balanced repeated replication method. | | |

Online Resource 2: Latent profile analysis mean estimates in comparison to the sample mean and corresponding p-values for profiles including market and country foods

|  | **Market Food Dominant** | | **Country Food Dominant** | | **Diverse Consumption** | | **Low  Consumption** | |
| --- | --- | --- | --- | --- | --- | --- | --- | --- |
| **Food item/category** | **Mean Estimate** | **p** | **Mean Estimate** | **p** | **Mean Estimate** | **p** | **Mean Estimate** | **p** |
| **Country foods** |  |  |  |  |  |  |  |  |
| Dried beluga | -0.26 | <0.001 | 0.88 | <0.001 | 0.31 | 0.01 | -0.21 | <0.001 |
| Beluga meat | -0.27 | <0.001 | 1.12 | <0.001 | 0.19 | 0.18 | -0.18 | <0.001 |
| Beluga blubber | -0.24 | <0.001 | 0.73 | <0.001 | 0.27 | 0.003 | -0.16 | 0.04 |
| Beluga mattaaq | -0.28 | <0.001 | 0.83 | <0.001 | 0.35 | 0.006 | -0.20 | 0.005 |
| Seal meat | -0.24 | <0.001 | 1.54 | <0.001 | -0.02 | 0.77 | -0.18 | 0.01 |
| Seal blubber | -0.20 | <0.001 | 1.18 | <0.001 | 0.04 | 0.64 | -0.19 | 0.004 |
| Seal liver | -0.25 | <0.001 | 1.62 | <0.001 | -0.07 | 0.36 | -0.14 | 0.05 |
| Dried Caribou | -0.33 | <0.001 | 0.76 | <0.001 | 0.59 | <0.001 | -0.34 | <0.001 |
| Caribou meat | -0.31 | <0.001 | 0.74 | <0.001 | 0.64 | <0.001 | -0.41 | <0.001 |
| Ptarmigan | -0.22 | <0.001 | 0.88 | <0.001 | 0.16 | 0.11 | -0.13 | 0.04 |
| Goose | -0.30 | <0.001 | 1.19 | <0.001 | 0.25 | 0.02 | -0.22 | 0.002 |
| Wild bird eggs | -0.29 | <0.001 | 1.18 | <0.001 | 0.16 | 0.18 | -0.13 | 0.04 |
| Dried fish | -0.38 | <0.001 | 0.97 | <0.001 | 0.53 | <0.001 | -0.28 | <0.001 |
| Lake trout | -0.26 | <0.001 | 0.99 | <0.001 | 0.25 | 0.02 | -0.21 | <0.001 |
| Sea trout | -0.18 | <0.001 | 0.73 | 0.001 | 0.30 | 0.004 | -0.28 | <0.001 |
| Arctic char | -0.35 | <0.001 | 0.94 | <0.001 | 0.44 | <0.001 | -0.21 | 0.01 |
| Mollusks | -0.30 | <0.001 | 1.05 | <0.001 | 0.26 | 0.04 | -0.15 | 0.02 |
| Seaweed | -0.23 | <0.001 | 0.81 | <0.001 | 0.23 | 0.06 | -0.16 | 0.01 |
| Wildberries | -0.24 | <0.001 | 0.53 | 0.002 | 0.52 | <0.001 | -0.33 | <0.001 |
| Bannock | -0.30 | <0.001 | 0.87 | <0.001 | 0.36 | <0.001 | -0.19 | 0.02 |
| Suuvalik | -0.33 | <0.001 | 0.57 | <0.001 | 0.56 | <0.001 | -0.21 | 0.01 |
| **Market foods** |  |  |  |  |  |  |  |  |
| Canned fruit | -0.15 | 0.008 | 0.24 | 0.12 | 0.61 | <0.001 | -0.47 | <0.001 |
| Fruit puree | -0.23 | <0.001 | 0.37 | 0.05 | 0.59 | 0.003 | -0.35 | <0.001 |
| Fruits | 0.08 | 0.27 | 0.24 | 0.09 | 0.59 | <0.001 | -0.86 | <0.001 |
| Vegetables | 0.12 | 0.09 | 0.26 | 0.13 | 0.58 | <0.001 | -0.93 | <0.001 |
| Potatoes | 0.16 | 0.003 | -0.07 | 0.62 | 0.55 | <0.001 | -0.84 | <0.001 |
| Tomatoes | 0.04 | 0.53 | 0.10 | 0.51 | 0.38 | 0.005 | -0.51 | <0.001 |
| Legumes/Nuts | -0.05 | 0.46 | 0.32 | 0.03 | 0.46 | <0.001 | -0.53 | <0.001 |
| Red meat | 0.04 | 0.51 | 0.06 | 0.76 | 0.64 | <0.001 | -0.78 | <0.001 |
| Poultry | 0.07 | 0.27 | 0.14 | 0.27 | 0.48 | <0.001 | -0.69 | <0.001 |
| Canned fish | -0.11 | 0.02 | 0.30 | 0.05 | 0.36 | 0.004 | -0.29 | <0.001 |
| Processed meats | -0.01 | 0.89 | 0.32 | 0.09 | 0.52 | <0.001 | -0.66 | <0.001 |
| Bacon | -0.05 | 0.32 | 0.37 | 0.003 | 0.46 | <0.001 | -0.55 | <0.001 |
| Eggs | 0.10 | 0.07 | 0.10 | 0.51 | 0.38 | <0.001 | -0.63 | <0.001 |
| Dairy products | 0.17 | 0.003 | 0.37 | <0.001 | 0.38 | <0.001 | -0.88 | <0.001 |
| Wholegrains | -0.03 | 0.60 | 0.34 | 0.01 | 0.45 | <0.001 | -0.55 | <0.001 |
| Refined grains | 0.21 | <0.001 | 0.33 | <0.001 | 0.43 | <0.001 | -0.97 | <0.001 |
| Snacks | 0.05 | 0.37 | 0.30 | 0.06 | 0.47 | <0.001 | -0.72 | <0.001 |
| Popcorn | -0.07 | 0.13 | 0.10 | 0.48 | 0.40 | 0.006 | -0.33 | <0.001 |
| Sugary foods | -0.002 | 0.97 | 0.33 | 0.04 | 0.47 | <0.001 | -0.63 | <0.001 |
| Coffee | 0.09 | 0.05 | -0.10 | 0.44 | 0.02 | 0.80 | -0.14 | 0.10 |
| Tea | -0.06 | 0.33 | 0.22 | 0.17 | 0.08 | 0.36 | -0.07 | 0.33 |
| Carbonated beverages | 0.01 | 0.83 | -0.05 | 0.69 | 0.19 | 0.01 | -0.20 | 0.02 |
| Fruit juices | 0.03 | 0.59 | 0.18 | 0.13 | 0.38 | <0.001 | -0.53 | <0.001 |
| Other beverages | -0.11 | 0.03 | 0.34 | 0.03 | 0.31 | 0.01 | -0.28 | <0.001 |
| Estimate means above the sample mean are highlighted in blue.  Estimate means below the sample mean are highlighted in red.  Statistically significant estimate means (p values<0.05) are highlighted in grey. | | | | | | | | |

Online Resource 3: Latent profile analysis mean estimates in comparison to the sample mean and corresponding p-values for profiles including country foods only

|  | **None** | | **Low** | | **Moderate** | | **High** | |
| --- | --- | --- | --- | --- | --- | --- | --- | --- |
| **Food item/category** | **Mean Estimate** | **p** | **Mean Estimate** | **p** | **Mean Estimate** | **p** | **Mean Estimate** | **p** |
| Dried beluga | -0.49 | <0.001 | 0.01 | 0.91 | 0.55 | 0.09 | 0.78 | <0.001 |
| Beluga meat | -0.36 | <0.001 | -0.05 | 0.70 | 0.31 | 0.38 | 1.04 | <0.001 |
| Beluga blubber | -0.54 | <0.001 | 0.15 | 0.30 | 0.28 | 0.09 | 0.78 | <0.001 |
| Beluga mattaaq | -0.51 | <0.001 | -0.01 | 0.93 | 0.63 | 0.01 | 0.81 | <0.001 |
| Seal meat | -0.48 | <0.001 | 0.05 | 0.82 | 0.00 | 0.99 | 1.60 | <0.001 |
| Seal blubber | -0.39 | <0.001 | 0.05 | 0.89 | -0.08 | 0.71 | 1.38 | <0.001 |
| Seal liver | -0.41 | <0.001 | -0.01 | 0.96 | -0.17 | 0.19 | 1.92 | <0.001 |
| Dried Caribou | -0.66 | <0.001 | -0.07 | 0.74 | 1.18 | <0.001 | 0.61 | 0.008 |
| Caribou meat | -0.67 | <0.001 | -0.03 | 0.90 | 1.10 | <0.001 | 0.61 | <0.001 |
| Ptarmigan | -0.40 | <0.001 | 0.10 | 0.53 | -0.04 | 0.84 | 1.07 | <0.001 |
| Goose | -0.50 | <0.001 | -0.03 | 0.77 | 0.49 | 0.05 | 1.15 | <0.001 |
| Wild bird eggs | -0.46 | <0.001 | -0.02 | 0.86 | 0.33 | 0.38 | 1.22 | <0.001 |
| Dried fish | -0.62 | <0.001 | -0.06 | 0.69 | 0.95 | <0.001 | 0.86 | <0.001 |
| Lake trout | -0.42 | <0.001 | -0.02 | 0.83 | 0.38 | 0.25 | 0.98 | <0.001 |
| Sea trout | -0.32 | <0.001 | -0.03 | 0.82 | 0.26 | 0.39 | 0.85 | <0.001 |
| Arctic char | -0.67 | <0.001 | 0.02 | 0.88 | 0.81 | <0.001 | 0.90 | <0.001 |
| Mollusks | -0.50 | <0.001 | -0.04 | 0.67 | 0.60 | 0.15 | 1.00 | <0.001 |
| Seaweed | -0.40 | <0.001 | 0.04 | 0.78 | 0.25 | 0.65 | 0.84 | 0.008 |
| Wildberries | -0.49 | <0.001 | 0.01 | 0.94 | 0.70 | <0.001 | 0.49 | <0.001 |
| Bannock | -0.49 | <0.001 | 0.05 | 0.74 | 0.41 | 0.01 | 0.85 | <0.001 |
| Suuvalik | -0.54 | <0.001 | 0.03 | 0.81 | 0.76 | <0.001 | 0.49 | 0.01 |
| Estimate means above the sample mean are highlighted in blue.  Estimate means below the sample mean are highlighted in red.  Statistically significant estimate means (p values<0.05) are highlighted in grey. | | | | | | | | |


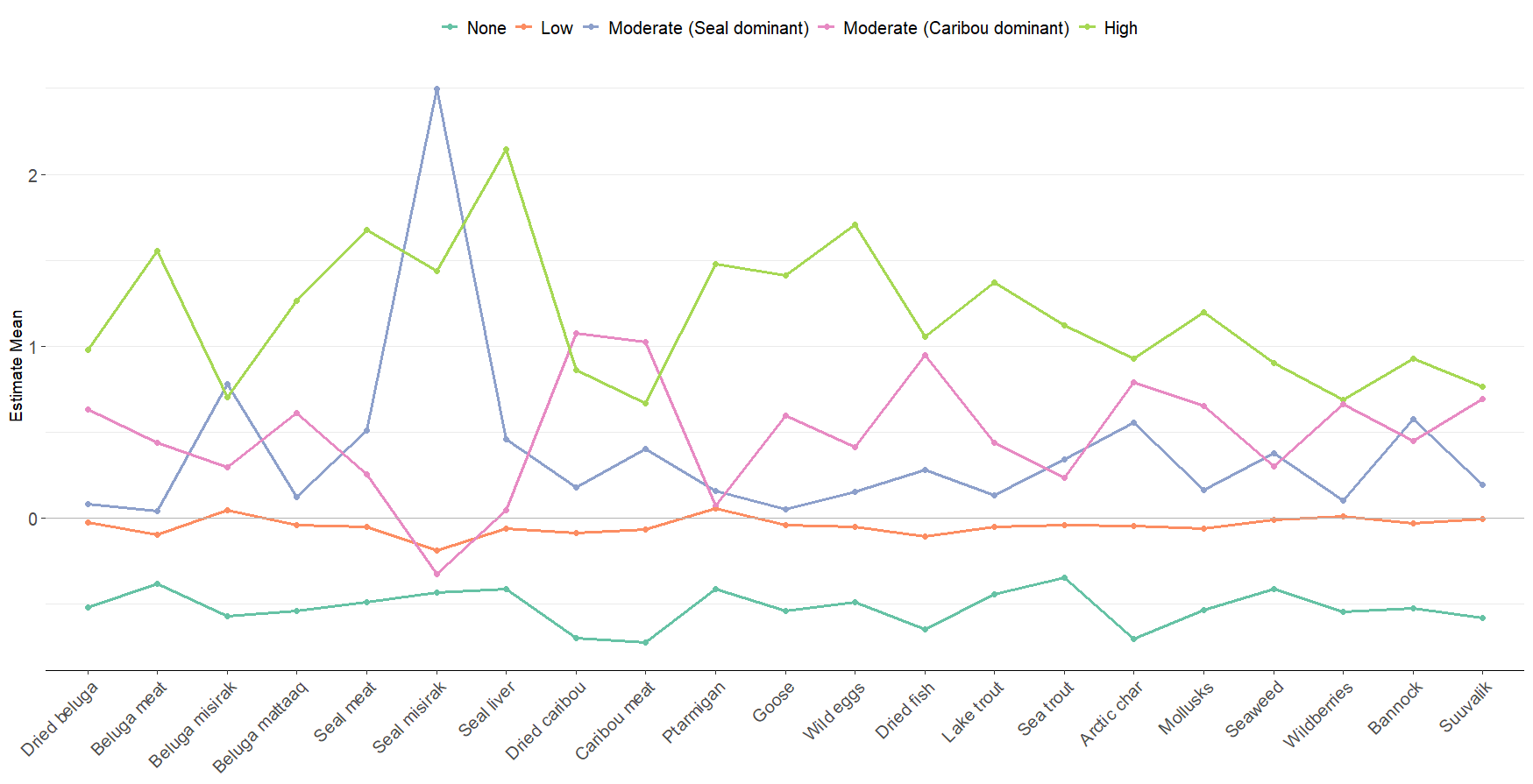


Online Resource 4: Dietary profiles using country food variables only with 5 profiles. Sample mean represented by “zero” line.
